# Supplementary material for: Molecular mechanisms that stabilize short term synaptic plasticity during presynaptic homeostatic plasticity
Source: eLife. 2018 Nov 13;7:e40385. doi: 10.7554/eLife.40385 (PMC6250423; doi:10.7554/eLife.40385)
Supplement: Supplementary file 1. [file elife-40385-supp1.docx]

| **Supplementary file 1.**  **Supplemental Table 1.**  Summary of raw data | | |  |  |  |  |  |  |  |  |  |  |
| --- | --- | --- | --- | --- | --- | --- | --- | --- | --- | --- | --- | --- |
|  |  |  |  |  |  |  |  |  |  |  |  |  |
| **Figure** | **Genotype** | **[Ca^2+^]_e_  (mM)** | **Condition (-/+ PhTx)** | **mEPSP amplitude (mV)** | **EPSP amplitude (mV)** | **Quantal Content** | **EPSC amplitude (nA)** | **Cum. EPSC amplitude (nA)** | **RRP (vesicles)** | **Fold Change (+/- PdBu)** | **Fold Change 4PR (+/- PdBu)** | **n** |
| 1E | *w^1118^* (- PdBu) | 0.75 | - | 0.71±0.05 |  | 235.1±29.0 | 154.2±12.6 |  |  |  |  | 19 |
|  | *w^1118^* (+ PdBu) | 0.75 | - | 0.73±0.03 |  | 320.8±24.6 | 227.0±12.4 |  |  | 1.47±0.08 |  | 10 |
|  | *w^1118^* (- PdBu) | 0.75 | + | 0.37±0.02 |  | 397.0±42.5 | 142.9±14.7 |  |  |  |  | 9 |
|  | *w^1118^* (+ PdBu) | 0.75 | + | 0.34±0.01 |  | 638.8±57.5 | 211.0±15.0 |  |  | 1.48±0.11 |  | 10 |
|  |  |  |  |  |  |  |  |  |  |  |  |  |
| 2B-D | *w^1118^* | 0.3 | - | 0.92±0.04 | 29.6±1.1 | 32.8±1.2 |  |  |  |  |  | 14 |
|  | *w^1118^* | 0.3 | + | 0.39±0.02 | 26.7±1.2 | 68.4±3.0 |  |  |  |  |  | 12 |
|  | *Df ^Rop^/+* | 0.3 | - | 0.84±0.05 | 32.1±1.6 | 38.7±1.8 |  |  |  |  |  | 13 |
|  | *Df ^Rop^/+* | 0.3 | + | 0.47±0.02 | 24.2±2.6 | 52.6±5.7 |  |  |  |  |  | 12 |
| 2E-H, 8A-D | *w^1118^* | 0.3 | - | 0.83±0.02 | 27.3±0.7 | 33.9±1.0 |  |  |  |  |  | 58 |
|  | *w^1118^* | 0.3 | + | 0.39±0.02 | 25.0±1.8 | 64.2±3.4 |  |  |  |  |  | 22 |
|  | *Rop^G27^/+* | 0.3 | - | 0.90±0.05 | 26.3±1.5 | 30.1±1.6 |  |  |  |  |  | 24 |
|  | *Rop^G27^/+* | 0.3 | + | 0.41±0.02 | 16.4±0.9 | 42.6±3.4 |  |  |  |  |  | 20 |
|  | *syx1A****^Δ^****^229^/+* | 0.3 | - | 0.82±0.04 | 24.1±1.4 | 30.3±1.8 |  |  |  |  |  | 23 |
|  | *syx1A****^Δ^****^229^/+* | 0.3 | + | 0.43±0.03 | 22.2±2.2 | 51.5±3.4 |  |  |  |  |  | 19 |
|  | *Rop^G27^/syx1A* ***^Δ^****^229^* | 0.3 | - | 0.99±0.05 | 25.6±1.4 | 26.2±1.5 |  |  |  |  |  | 28 |
|  | *Rop^G27^/syx1A* ***^Δ^****^229^* | 0.3 | + | 0.47±0.02 | 23.1±2.0 | 47.6±4.0 |  |  |  |  |  | 17 |
|  |  |  |  |  |  |  |  |  |  |  |  |  |
| 3A-D | *c155-GAL4/+* | 0.3 | - | 0.82±0.06 | 27.0±2.1 | 33.1±1.6 |  |  |  |  |  | 10 |
|  | *c155-GAL4/+* | 0.3 | + | 0.38±0.03 | 23.3±1.3 | 64.6±7.0 |  |  |  |  |  | 8 |
|  | *c155-GAL4/Rop-RNAi* | 0.3 | - | 0.75±0.06 | 19.5±1.3 | 28.3±2.1 |  |  |  |  |  | 24 |
|  | *c155-GAL4/Rop-RNAi* | 0.3 | + | 0.36±0.02 | 13.9±1.3 | 39.9±3.7 |  |  |  |  |  | 19 |
|  |  |  |  |  |  |  |  |  |  |  |  |  |
| 4A-C, 4F | *w^1118^* | 1.5 | - | 0.73±0.04 |  |  | 191.5±9.8 |  |  |  |  | 33 |
|  | *w^1118^* | 1.5 | + | 0.40±0.01 |  |  | 183.6±13.5 |  |  |  |  | 9 |
|  | *Rop^G27^/+* | 1.5 | - | 0.64±0.03 |  |  | 146.8±9.8 |  |  |  |  | 20 |
|  | *Rop^G27^/+* | 1.5 | + | 0.35±0.02 |  |  | 109.2±5.3 |  |  |  |  | 8 |
|  | *rim^103^/+* | 1.5 | - | 0.75±0.08 |  |  | 175.6±13.3 |  |  |  |  | 10 |
|  | *rim^103^/+* | 1.5 | + | 0.44±0.02 |  |  | 147.6±11.3 |  |  |  |  | 12 |
|  | *Rop^G27^/rim^103^* | 1.5 | - | 0.80±0.05 |  |  | 156.4±21.1 |  |  |  |  | 9 |
|  | *Rop^G27^/rim^103^* | 1.5 | + | 0.49±0.03 |  |  | 86.5±7.5 |  |  |  |  | 14 |
| 4D-F | *w^1118^* | 0.75 | - | 0.59±0.04 |  |  | 103.3±7.5 |  |  |  |  | 9 |
|  | *Rop^G27^/+* | 0.75 | - | 0.55±0.04 |  |  | 57.1±4.6 |  |  |  |  | 10 |
|  | *w^1118^* | 3.0 | - | 0.64±0.04 |  |  | 281.7±17.7 |  |  |  |  | 20 |
|  | *w^1118^* | 3.0 | + | 0.28±0.02 |  |  | 229.1±16.3 |  |  |  |  | 11 |
|  | *Rop^G27^/+* | 3.0 | - | 0.58±0.05 |  |  | 208.6±11.8 |  |  |  |  | 15 |
|  | *Rop^G27^/+* | 3.0 | + | 0.32±0.04 |  |  | 149.9±17.1 |  |  |  |  | 12 |
|  |  |  |  |  |  |  |  |  |  |  |  |  |
|  |  |  |  |  |  |  |  |  |  |  |  |  |
|  |  |  |  |  |  |  |  |  |  |  |  |  |
| 5A-C | *w^1118^* | 1.5 | - | 0.70±0.03 |  |  |  | 697±54 | 1017±97 |  |  | 11 |
|  | *w^1118^* | 1.5 | + | 0.40±0.03 |  |  |  | 745±64 | 1929±167 |  |  | 14 |
|  | *Rop^G27^/+* | 1.5 | - | 0.80±0.06 |  |  |  | 764±91 | 1099±218 |  |  | 15 |
|  | *Rop^G27^/+* | 1.5 | + | 0.46±0.03 |  |  |  | 962±109 | 2247±358 |  |  | 10 |
|  |  |  |  |  |  |  |  |  |  |  |  |  |
| 6 | *w^1118^* | 0.3 | - | 0.86±0.02 | 28.9±0.6 | 34.8±0.8 |  |  |  |  |  | 91 |
|  | *w^1118^* | 0.3 | + | 0.43±0.01 | 26.0±1.2 | 61.1±2.6 |  |  |  |  |  | 42 |
|  | *Rop^G27^/+* | 0.3 | - | 0.90±0.05 | 26.3±1.5 | 30.1±1.6 |  |  |  |  |  | 24 |
|  | *Rop^G27^/+* | 0.3 | + | 0.41±0.02 | 16.4±0.9 | 42.6±3.4 |  |  |  |  |  | 20 |
|  | *rim^103^/+* | 0.3 | - | 0.87±0.03 | 27.5±1.1 | 32.3±1.4 |  |  |  |  |  | 38 |
|  | *rim^103^/+* | 0.3 | + | 0.43±0.02 | 20.8±1.4 | 48.4±2.6 |  |  |  |  |  | 26 |
|  | *Rop^G27^/rim^103^* | 0.3 | - | 0.89±0.04 | 16.7±1.3 | 18.7±1.3 |  |  |  |  |  | 34 |
|  | *Rop^G27^/rim^103^* | 0.3 | + | 0.46±0.01 | 9.0±0.9 | 20.5±2.0 |  |  |  |  |  | 49 |
|  | *w^1118^* | 0.3 | - | 0.84±0.02 | 28.2 0.7 | 34.7 1.1 |  |  |  |  |  | 74 |
|  | *w^1118^* | 0.3 | + | 0.40±0.02 | 24.8±1.5 | 62.1 3.3 |  |  |  |  |  | 25 |
|  | *rbp^STOP1^/+* | 0.3 | - | 0.82±0.04 | 28.0±2.4 | 34.2 2.3 |  |  |  |  |  | 11 |
|  | *rbp^STOP1^/+* | 0.3 | + | 0.47±0.02 | 30.5±1.5 | 66.4 4.5 |  |  |  |  |  | 9 |
|  | *Rop^G27^/rbp^STOP1^* | 0.3 | - | 0.87±0.04 | 24.4±2.4 | 28.2 2.7 |  |  |  |  |  | 14 |
|  | *Rop^G27^/rbp^STOP1^* | 0.3 | + | 0.43±0.03 | 18.0±2.0 | 43.9 6.1 |  |  |  |  |  | 11 |
|  | *dunc-13^P84200^/+* | 0.3 | - | 0.91±0.04 | 29.0±1.1 | 32.5 1.7 |  |  |  |  |  | 12 |
|  | *dunc-13^P84200^/+* | 0.3 | + | 0.47±0.02 | 25.1±1.1 | 54.0 3.1 |  |  |  |  |  | 9 |
|  | *rim^103^/+; dunc-13^P84200^* | 0.3 | - | 1.03±0.09 | 24.8±2.2 | 25.4 3.0 |  |  |  |  |  | 11 |
|  | *rim^103^/+; dunc-13^P84200^* | 0.3 | + | 0.45±0.03 | 21.7±1.7 | 49.9 4.8 |  |  |  |  |  | 12 |
|  | *rim^103^/rbp^STOP1^* | 0.3 | - | 0.83±0.05 | 18.2±1.5 | 22.3 2.0 |  |  |  |  |  | 11 |
|  | *rim^103^/rbp^STOP1^* | 0.3 | + | 0.47±0.03 | 19.5±3.5 | 40.4 5.8 |  |  |  |  |  | 11 |
|  |  |  |  |  |  |  |  |  |  |  |  |  |
| 7B | *w^1118^* (+ PdBu) | 0.75 | - |  |  |  |  |  |  | 1.66±0.20 |  | 8 |
|  | *Rop^G27^/rim^103^* (+ PdBu) | 0.75 | - |  |  |  |  |  |  | 3.62±0.55 |  | 7 |
| 7C, 7D | *w^1118^* (+ PdBu) | 0.75 | - |  |  |  |  |  |  | 1.80±0.13 | 0.82±0.03 | 13 |
|  | *Rop^G27^/+* (+ PdBu) | 0.75 | - |  |  |  |  |  |  | 1.88±0.07 | 0.74±0.03 | 8 |
|  | *rim^103^/+* (+ PdBu) | 0.75 | - |  |  |  |  |  |  | 1.87±0.14 | 0.79±0.04 | 8 |
|  | *Rop^G27^/rim^103^* (+ PdBu) | 0.75 | - |  |  |  |  |  |  | 3.62±0.55 | 0.49±0.02 | 8 |
|  |  |  |  |  |  |  |  |  |  |  |  |  |
| 9 | *w^1118^* | 1.5 | - | 0.73±0.05 |  |  | 217.4±7.3 |  |  |  |  | 29 |
|  | *w^1118^* | 1.5 | + | 0.39±0.02 |  |  | 204.1±8.5 |  |  |  |  | 23 |
|  | *Rop^G11^/+* | 1.5 | - | 0.77±0.10 |  |  | 178.0±16.2 |  |  |  |  | 12 |
|  | *Rop^G11^/+* | 1.5 | + | 0.33±0.02 |  |  | 149.8±11.2 |  |  |  |  | 19 |
|  | *Rop^G27^/Rop^G11^* | 1.5 | - | 0.90±0.08 |  |  | 134.2±12.2 |  |  |  |  | 19 |
|  | *Rop^G27^/Rop^G11^* | 1.5 | + | 0.42±0.02 |  |  | 115.1±12.4 |  |  |  |  | 13 |
|  | *Df ^Rop^/Rop^G11^* | 1.5 | - | 0.73±0.07 |  |  | 156.3±17.2 |  |  |  |  | 10 |
|  | *Df ^Rop^/Rop^G11^* | 1.5 | + | 0.46±0.02 |  |  | 149.3±15.4 |  |  |  |  | 9 |
